# Elevated inorganic carbon and salinity enhances photosynthesis and ATP synthesis in picoalga *Picocystis salinarum* as revealed by label free quantitative proteomics

**DOI:** 10.3389/fmicb.2023.1059199

**Published:** 2023-03-03

**Authors:** Jyoti Singh, Shubham Kaushik, Chinmaya Maharana, Gagan Deep Jhingan, Dolly Wattal Dhar

**Affiliations:** ^1^Centre for Conservation and Utilization of Blue Green Algae, Division of Microbiology, Indian Agricultural Research Institute, New Delhi, India; ^2^Department of Earth Sciences, Pondicherry University, Puducherry, India; ^3^Vproteomics, Valerian Chem Private Limited, New Delhi, India; ^4^Water Technology Centre, Indian Agricultural Research Institute, New Delhi, India; ^5^School of Agricultural Sciences, Sharda University, Greater Noida, Uttar Pradesh, India

**Keywords:** *Picocystis salinarum*, proteomics, haloalkaliphilic, algae, label free quantitation (LFQ), soda lake, extremophile

## Abstract

Saline soda lakes are of immense ecological value as they niche some of the most exclusive haloalkaliphilic communities dominated by bacterial and archaeal domains, with few eukaryotic algal representatives. A handful reports describe *Picocystis* as a key primary producer with great production rates in extremely saline alkaline habitats. An extremely haloalkaliphilic picoalgal strain, *Picocystis salinarum* SLJS6 isolated from hypersaline soda lake Sambhar, Rajasthan, India, grew robustly in an enriched soda lake medium containing mainly Na_2_CO_3_, 50 g/l; NaHCO_3,_ 50 g/l, NaCl, 50 g/l (salinity ≈150‰) at pH 10. To elucidate the molecular basis of such adaptation to high inorganic carbon and NaCl concentrations, a high-throughput label-free quantitation based quantitative proteomics approach was applied. Out of the total 383 proteins identified in treated samples, 225 were differentially abundant proteins (DAPs), of which 150 were statistically significant (*p* < 0.05) including 70 upregulated and 64 downregulated proteins after 3 days of growth in highly saline-alkaline medium. Most DAPs were involved in photosynthesis, oxidative phosphorylation, glucose metabolism and ribosomal structural components envisaging that photosynthesis and ATP synthesis were central to the salinity-alkalinity response. Key components of photosynthetic machinery like photosystem reaction centres, adenosine triphosphate (ATP) synthase ATP, Rubisco, Fructose-1,6-bisphosphatase, Fructose-bisphosphate aldolase were highly upregulated. Enzymes peptidylprolyl isomerases (PPIase), important for correct protein folding showed remarkable marked-up regulation along with other chaperon proteins indicating their role in osmotic adaptation. Enhanced photosynthetic activity exhibited by *P. salinarum* in highly saline-alkaline condition is noteworthy as photosynthesis is suppressed under hyperosmotic conditions in most photosynthetic organisms. The study provided the first insights into the proteome of extremophilic alga *P. salinarum* exhibiting extraordinary osmotic adaptation and proliferation in polyextreme conditions prevailing in saline sodic ecosystems, potentially unraveling the basis of resilience in this not so known organism and paves the way for a promising future candidate for biotechnological applications and model organism for deciphering the molecular mechanisms of osmotic adaptation. The mass spectrometry proteomics data is available at the ProteomeXchange Consortium *via* the PRIDE partner repository with the dataset identifier PXD037170.

## 1. Introduction

Soda lakes and pans are naturally occurring extreme environments characterized by high alkalinity and salinity, mostly shallow, formed in closed drainage basins exposed to high evaporation rates (Jones et al., 1998). Although common, their occurrence is less widespread than other saline waters. Soda lakes have saline water dominated by sodium and carbonate species, typically exceeding a pH of 9. What clearly distinguishes them from other saline waters is the low concentrations of alkaline earth cations Ca^2+^ and Mg^2+^ that allows the water to become enriched with CO_3_^2−^ and Cl^−^, with pH > 9. They represent the most stable high-pH environments on Earth (Jones et al., 1998; Grant, 2004; Warren, 2006). Moreover, soda lakes are interesting from evolutionary and geobiological point of view. There are records of fossil Soda lakes as old as 2.3 billion years ago (Karpeta, 1989). Soda lakes are supposed to be habitats of relict microbial communities, Zavarzin (1993), has reasoned credibly that alkaline soda lakes have ancient microbial communities as they exhibit remarkable prokaryotic diversity and metabolic diversity that is sufficient to maintain an autonomous microbial community, and therefore are speculated as the origin of prokaryotic diversity. The “Precambrian explosion” of prokaryote diversity might have taken place in alkaline environments (Jones et al., 1998). The Archaean ocean did not resemble the present day Na-Cl ocean and was probably dominated by Na-Cl-HCO_3_ (Maisonneuve, 1982; Kempe and Degens, 1985). According to the controversial “soda ocean hypothesis,” the chemistry of the early Archaean hydrosphere might had been similar to that of present day soda lakes (Warren, 2006). Soda lakes represents the most productive aquatic environments in the world, especially the more diluted ones (Grant, 2004), being “polyextreme ecosystems” harboring some of the most exclusive haloalkaliphilic communities on Earth performing special biogeochemical cycling, dominated by bacterial and archaeal domains (Jones et al., 1998; Zavarzin et al., 1999; Grant, 2004; Sorokin et al., 2014, 2015). Oxyphotobacteria (photosynthetic cyanobacteria) as well as anoxygenic photoautotrophic bacteria, are often the key players responsible for primary production (Grant, 2004). However, our knowledge of the diversity and understanding of the biogeochemical functioning of these systems is still incomplete (Grant, 2004; Tweedley et al., 2019; Saccò et al., 2021). The microbial ecology of very hypersaline soda lakes (e.g., Lakes Magadi, Natron in Africa and Lake Sambhar, India) is unique and dominated by alkaliphilic haloarchaea that are well characterized now, but the knowledge of diversity of oxygenic primary producers (cyanobacteria and algae) is limited. Among cyanobacteria, the *Spirulina– Cyanospira–Arthrospira* clade is well established in tropical soda lakes (Krienitz et al., 2013), while information on others is limited (Sorokin et al., 2015). *Ctenocladus, Picocystis salinarium and Dunaliella* are known representative of green algae in Soda lakes (Samylina et al., 2014). For detailed account of aerobic and anaerobic processes and microbes in soda lakes, reviews by Zavarzin and coworkers (Zavarzin, 1993; Zhilina and Zavarzin, 1994; Zavarzin et al., 1999) and Grant and coworkers (Grant et al., 1990; Jones et al., 1994, 1998; Grant and Jones, 2000) should be referred. Recent comprehensive reviews by Sorokin et al. (2014, 2015) may be referred for microbiological studies on Soda lakes.

Soda lake prokaryotes have attracted immense scientific attention for their biotechnological potential as the exo-enzymes produced by them are expected to be active and stable at high pH and high salt concentration (Zhilina and Zavarzin, 1994; Grant, 2004; Sorokin et al., 2015; Sousa et al., 2015). Haloalkaliphilic anaerobic microbial communities have found promising application in the fermentation of lignocellulosic feedstocks material subjected to an alkaline pre-treatment, methane production and sulfur removal technology (Sorokin et al., 2015; Sousa et al., 2015). Among microalgae, prokaryotic cyanobacterial strains *Arthrospira (Spirulina) platensis* (Kishi and Toda, 2018; Ye et al., 2020), *Euhalothece* sp. isolated from soda lake have been shown to be suitable for bicarbonate based integrated carbon capture production system (Mikhodyuk et al., 2008; Gerasimenko and Mikhodyuk, 2009; Chi et al., 2013; Zhu et al., 2018).

*Picocystis salinarum* Lewin (Lewin et al., 2000), is a unique green alga characterized just two decades ago, inhabiting haloalkaline systems, with a separate lineage within the prasinophyte. The cells are spherical and non-flagellated, 2–3 μm in size, and display a distinct set of pigments consisting of carotenoids alloxanthin, diatoxanthin, and monadoxanthin along with chlorophyll a and b. *P. salinarum* was first reported from a saline pond (100‰ salinity) at the San Francisco Salt Works, California, United States (Lewin et al., 2000; Lopes dos Santos et al., 2016). Since then, different *Picocystis* strains have been reported from extremely saline waterbodies, saline-soda lakes across the four continents, flourishing in extreme environmental conditions of pH up to 11, salinity up to 300‰ and temperature up to 60°C (Krienitz et al., 2012; Ali et al., 2017; Ouada et al., 2018; Pálmai et al., 2020). Krienitz et al. (2012) states that “*Picocystis salinarum* from saline inland waters represents a link between marine and freshwater habitats from both an ecological and a phylogenetic point of view and is therefore of great interest.” Also, recent reports have suggested that *Picocystis* may have potential biotechnological applications from wastewater treatment (Ali et al., 2017; Ouada et al., 2018), to biofuel production (Wang et al., 2014; Delgado et al., 2021). In spite of demonstrating such unique environmental adaptations, flourishing and out competing other primary producers at extreme salinities and influencing the food web dynamics (Krienitz and Kotut, 2010), *P. salinarum* has gone unreported in most of the culture independent metagenomic studies of saline ecosystems all over, indicating the lack of information about this organism on molecular level. Only a few studies highlight the ecophysiology of this organism in such harsh environmental conditions. Adaptation to low light, with good light utilization along wide temperature gradients and accumulation of osmolytes are key adaptations (Roesler et al., 2002; Pálmai et al., 2020). *Picocystis* strain ML from Mono Lake was observed to grow over salinity range of 0–260‰, and was reported to contain dimethylsulfoniopropionate (DMSP) and glycine betaine (GB) as osmolytes. Also, regulation of cell size and cytoplasmic volume contributed to balance the osmotic stress (Roesler et al., 2002). Nevertheless, an organism with such capabilities is worthy of extensive studies.

Recently, an extremely haloalkaliphilic strain *P. salinarum* SLJS6 was isolated from brine water of the largest inland hypersaline soda lake of India, Lake Sambhar, Rajasthan, India. The strain exhibited vigorous growth in a culture medium containing mainly Na_2_CO_3_, 50 g/l; NaHCO_3,_ 50 g/l; NaCl, 50 g/l (salinity ≈150 ‰, DIC ≈ 1 M NaHCO_3_/Na_2_CO_3_) at pH 10 and was prospected to be promising for bicarbonate-based biomass production and carbon capture (Singh et al., 2022). It seemed interesting to find how the organism was able to do so. Therefore, in an attempt to identify and understand the underlying mechanisms of osmotic adaptation in *P. salinarum* SLJS6, the present study was undertaken.

Proteomics is a powerful tool that can aid the understanding of intricate biological processes by identifying and quantifying expressed proteins in cells, their modifications and their interactions. Proteome analysis during stress response can provide better insights than transcriptome, as changes in proteins reflect the real response to environmental stimuli. In order to study the relative differences in protein expression in complex biological samples, under a particular condition/time, quantitative MS-based proteomics that uses labeled or label-free proteins can be applied. In Mass spectrometry-based label-free quantitative proteomics, two approaches can be used (i) the measurements of changes in chromatographic ion intensity such as peptide peak areas or peak heights, (ii) spectral counting of the identified proteins (Patel et al., 2009). Both techniques have proven to be useful specially in studying proteins of low abundance (Dowle et al., 2016).

In the present study, a high-throughput label-free quantitation (LFQ) based quantitative proteomics approach was used to study the proteome of haloalkaliphilic algal strain *P. salinarum SLJS6* in response to high NaCl and inorganic carbon concentrations (salinity-alkalinity treatment). The data available on *P. salinarum* in protein repositories is limited as only two draft genomes are available till date. Nonetheless, we identified 225 differentially abundant proteins (DAPs), of which 150 were statistically significant (*p* < 0.05) including 70 upregulated and 64 downregulated proteins after 3 days of salt and alkalinity treatment. This study provided the first insights into proteome of extremophilic alga *P. salinarum* suggesting enhanced photosynthesis and ATP synthesis as the prime strategy to successfully thrive in highly saline-alkaline environments, potentially unraveling the underlying molecular basis of natronophily in this not so known organism which may be exploited for potential biotechnological use and as a model organism for deciphering the molecular mechanisms of osmotic adaptation. The results may also be interesting to physiologists and ecologists interested in eukaryotic phototrophic organisms thriving in highly haloalkaline ecosystems.

## 2. Materials and methods

### 2.1. Algal culture

*P. salinarum* SLJS6 (Genbank Accession no. MW750597) was previously isolated from brine water of hypersaline Sambhar soda lake and was found suitable for bicarbonate-based biomass production and carbon capture owing to its remarkable growth in an enriched soda lake medium (M medium) containing high concentrations dissolved inorganic carbon (DIC) in the form of carbonates and high salinity as NaCl (Singh et al., 2022). The algal cells were cultured in non-buffered BG-11 (Rippka, 1998) medium with 25 g/l NaCl and 16.8 g/l NaHCO_3_ at pH 8.5 and maintained at 30°C, 16:8 h light:dark cycle, under fluorescent light (50 μE s^−1^ m^−2^) for a month before the proteomics experiment. The culture was used as inoculum for a fresh batch of culture in the same medium under the same aforementioned conditions. From this fresh culture, the cells in the logarithmic phase (7 days after inoculation, OD = 0.7) were transferred to 50 ml of M medium (salinity-alkalinity treatment) and 50 ml of BG-11 medium (25 g/l NaCl, 16.8 g/l NaHCO_3,_ pH 8.5) in sterilized conical borosilicate flasks with three biological replicates of each control and treatment group. All the cultures were maintained at 30°C, with a 16:8 h light:dark cycle, under fluorescent light (50 μE s^−1^ m^−2^) for 3 days. The composition of the M medium was: NaHCO_3_ (50 g/l), Na_2_CO_3_ (50 g/l), NaCl (50 g/l), KCl (2 g/l), Na_2_SO_4_ (1.4 g/l), KNO_3_ (2.5 g/l), K_2_HPO_4_.3H_2_O (0.5 g/l); FeCl_3_ (0.0003 g/l), EDTA (0.0005 g/l), 1 ml of the A5 trace element solution (Mikhodyuk et al., 2008), pH 10. Algal culture in BG-11 medium with 25 g/l NaCl, 16.8 g/l NaHCO_3,_ pH 8.5 was taken as control. The rationale behind using BG-11 medium as a control for this study was that in our previous experiments, the other microalgal isolates from Sambhar soda lake preferred this BG-11 medium for profound growth whereas *P. salinarum* grew minimally in the respective medium. On the other hand, *P. salinarum* grew robustly in M medium while others showed declined growth. Therefore, Blue green algae growth medium could serve as an appropriate control for studying the osmotic adaptation of this extremophilic organism, mimicking the low salt condition that prevails in Lake Sambhar during monsoon season during which cyanobacterial blooms occur. After 3 days of growth in both control (BG-11) and treatment (M), algal cells were harvested by centrifugation at 10,000 ×*g* for 10 min at 4°C. and then washed with MiliQ thrice to remove any salts and later with phosphate buffered saline (PBS).

### 2.2. Protein extraction, quantification and sample preparation

The washed pellets were lysed in boiled hot 8 M Guanidine hydrochloride (GNHCL) plus 0.1 M TRIS pH 8.5 buffer. The cell lysis was performed by subjecting the samples to three cycles of sonication (30% amplitude for 30 s and 30-s rest between cycles). Samples were then centrifuged at 13,000 rpm for 10 min and the supernatant was collected and further used for LFQ proteomics experiments. The total protein concentration in each sample was quantified using Nanodrop One (Thermo Scientific).

### 2.3. Liquid chromatography–tandem mass spectrometry (LC–MS/MS) analysis

The protein samples extracted from the three biological replicates of both the control and treatment were used for LFQ-based quantitative proteomics analysis. 50 μg of each protein sample was first reduced using 5 mM tris (2-carboxyethyl) phosphine (TECP, Pierce) in 50 mM Ammonium Bicarbonate (ABC, pH ~ 8) at 37°C for 30 min and then alkylated using 50 mM Iodoacetamide (IAA, Sigma) in 50 mM ABC (pH ~ 8) in dark for another 30 min. After diluting the samples 10 times with water, the proteins were digested into smaller peptides using the serine protease Trypsin (Promega). This was added in the ratio of 1:50 (Trypsin: Lysate ratio) and incubated overnight at 37°C for complete digestion. The reaction was stopped by adding 10% trifluoroacetic acid (TFA). Digests were then cleaned up using Pierce™ C18 Spin Columns according to manufacturer’s protocol to remove any salts and dried using speed vacuum (Thermo Savant DNA 120). The pellet was finally resuspended in Buffer-A (0.1% formic acid in water). The clarified peptide digested samples (1 μg each) were then resolved on a 50-cm long EASY-Spray column (50 cm*75 μm) PepMap RSLC filled with 2 μm-C18 resin on nano1200 chromatography system (ThermoFischer Scientific) attached to QExactive mass spectrometer equipped with nano-electrospray ion source. The peptides were loaded with Buffer A and eluted with a 0–40% gradient of Buffer-B (80% acetonitrile, 0.1% formic acid) for 88 min, 40–95% gradient for 8 min, followed by 5% gradient for 4 min at a flow rate of 300 nl/min with a total run time of 100 min.

The QExactive spray voltage was set at 2.5 kV, S lens RF level at 50 and ITC heated capillary temperature at 275°C. The MS data were acquired in positive polarity using a data-dependent method choosing the 10 most intense peaks with charge state +2 to +5, exclude isotope option enabled and dynamic exclusion time of 12 s. MS1 spectra were acquired in the Orbitrap (Max IT = 100 ms, AGQ target = 1e6; R = 70 K, mass range = 350–1700; Profile data). Dynamic exclusion was employed for 12 s excluding all charge states for a given precursor. MS2 spectra were collected for top 10 peptides. MS2 (Max IT = 100 ms, R = 17.5 K, AGC target 1e5).

The MS1 or Full scan target was 1 × 106 with a maximum fill time of 100 ms with mass range set to 350–1700. Target value for MS2 or fragment scans was set at 1 × 105, and intensity threshold was set at 5 × 103. Isolation window of parent ion of interest was set at 2 m/z. Normalized collision energy for higher-energy collisional dissociation (HCD) was set at 27. Peptide match option was set to preferred mode along with activation of isotope exclusion option.

### 2.4. Analysis of differentially expressed proteins

#### 2.4.1. Protein identification and quantification

All samples were processed and RAW files MS/MS spectra generated were analyzed with Proteome Discoverer (v2.2) against the Uniprot *Picocystis salinarum* (3631 entries) proteome database^1^. The search engines Sequest HT and MS Amanda 2.0 based algorithms SEQUEST and AMANDA were used for matching peptides with proteins. For Sequest search, the precursor and fragment mass tolerances were set at 10 ppm and 0.02 Da, respectively. The protease used to generate peptides, i.e., enzyme specificity was set for trypsin/P (cleavage at the C terminus of “K/R: unless followed by “P”) along with maximum missed cleavages value of two. Carbamidomethyl on cysteine as fixed modification and oxidation of methionine and N-terminal acetylation was considered as variable modifications for database search. Minimum peptide length for search was set at 6, while maximum peptide length was set at 150. Both peptide spectrum match and protein false discovery rate were set to 0.01 FDR and determined using percolator node. Relative protein quantification was performed using Minora feature detector node of Proteome Discoverer 2.2 including a Minimum Trace Length of 5, Max. ΔRT of Isotope Pattern 0.2 min and considering only those with high peptide spectrum matches (PSM) confidence. The mass spectrometry proteomics data have been deposited to the ProteomeXchange Consortium *via* the PRIDE partner repository with the dataset identifier PXD037170.

### 2.5. Statistical analyses

Raw abundance values were used for the statistical analysis. Abundance values were log2 transformed followed by pre-processing or filtration. Proteins which were quantified in at least 2 replicates for each condition were filtered out and missing values were imputed on the basis of standard deviation. After filtration, the data was normalized followed by Student’s *t*-test to obtain significantly differentially expressed proteins between the two conditions. Significance was calculated based on *p*Value, i.e., *p*Value < 0.05. The log transformed abundance values were further Z scaled and used to plot Heatmap. All the significant proteins were used for data visualization using in-house R scripts (R packages used: ComplexHeatmap, RColorBrewer, randomcoloR, circlize, factoextra, FactoMineR, ggpubr, ggrepel, EnhancedVolcano, psych, corrplot).

### 2.6. Annotation of hypothetical proteins using BLAST algorithm

As many proteins were autoannotated as hypothetical proteins when identified against *Picocystis* database in Uniprot, the hypothetical proteins were subjected to NCBI protein BLAST using the followings parameters:

Enter query sequence:

- Fasta Sequences for all hypothetical proteins (243 proteins).

Choose search set:

- Database type: Standard databases (non-redundant).
- Database: UniProtKB/Swiss-Prot (swissprot).

Program selection:

- Algorithm: protein–protein BLAST.

Algorithm paramaters:

- Max target sequences - 100.
- Expect threshold - 0.05.

Scoring paramters:

- Matrix: BLOSSUM62.
- Gap Costs: Existence: 11 Extension: 1.
- Compositional adjustments: Conditional compositional score matrix adjustment.

BLAST results having maximum coverage and lowest E-value for proteins identified were selected.

## 3. Results

*P. salinarum* SLJS6 is a haloalkaliphilic strain, previously isolated from brine water of hypersaline Sambhar salt soda lake (Genbank Accession no. MW750597) and was found suitable for bicarbonate-based biomass production and carbon capture, owing to its remarkable growth in an enriched soda lake medium (M medium) containing mainly Na_2_CO_3_, 50 g/l, NaHCO_3,_ 50 g/l, NaCl, 50 g/l (salinity ≈150 ‰, DIC ≈ 1 M NaHCO_3_/Na_2_CO_3_) at pH 10. SLJS6 achieved a growth rate of 0.2282 d^−1^ and biomass productivity of 0.498 g/l/day when grown in M medium, which was almost >35 folds as compared to the growth achieved in BG-11 medium with 25 g/l NaCl and 16.8 g/l NaHCO_3_ at pH 8.5 (Singh et al., 2022). Concomitant increase in CO_2_ fixation rate (0.729 g/l/d) was also observed. In order to get insights into the underlying molecular mechanisms that are driving this extraordinary growth, carbon assimilation and osmotic adaptation, the proteomics study was carried out under the respective conditions of alkalinity and salinity in the two aforementioned growth media. The rationale behind the study was that the strain exhibited significantly faster growth rate in M medium (high DIC and NaCl) in comparison to BG-11 (low DIC and NaCl). It was hypothesized that the strain might be performing enhanced photosynthesis along with maintaining the osmotic balance to achieve vigorous growth.

### 3.1. Identification of DAPs in *Picocystis salinarum* using LFQ

**Figure 1:**
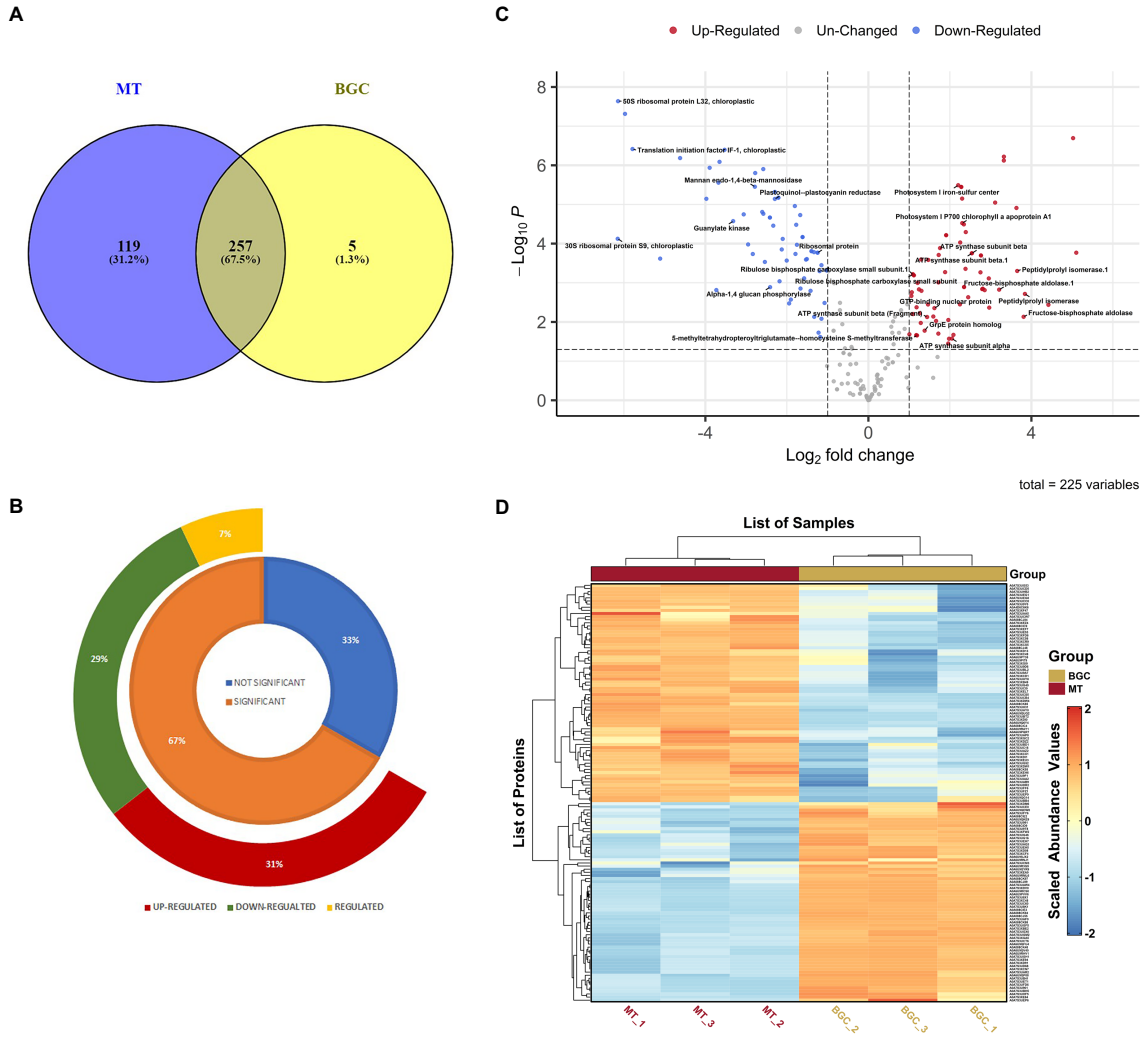
Differentially abundant proteins (DAPs) in elevated Ci and salt in *Picocystis salinarum* SLJS6. **(A)** Venn diagram of BG11 (BGC) and M (MT) specific proteins with overlapping regions indicating the number of common proteins. **(B)** doughnut chart highlighting the T-test results (the regulated proteins are those significantly altered proteins whose log2 Fold Change value fell between the cutoff values i.e., > − 1 and <1). **(C)** Volcano plot of the proteins quantified during LFQ analysis comparing control (BGC) to treatment (MT). Each point represents the difference in expression (fold-change) between the two groups plotted against the level of statistical significance. **(D)** Heatmap depicting DAPs comparing control (BGC) to treatment (MT) groups.

Three independent biological replicate cultures subjected to same ambient conditions of light and temperature were cultivated for the LFQ proteomics experiment and were treated uniformly throughout the experimental procedures. The proteomes of *P. salinarum* grown in M medium, subjected to high DIC and salinity were analyzed using LFQ and liquid chromatography–tandem mass spectrometry/ mass spectrometry (LC–MS/MS). The MS1 (mass range 350–2,000 m/z) and MS2 scans were acquired in Orbitrap Mass analyser with resolution of 70,000 and 17,500 at m/z 200–2,000, respectively. RAW files generated were analyzed with Proteome Discoverer (v2.2) against the Uniprot *Picocystis salinarum* (3,631 entries) proteome database and matched a total of 828 peptide groups and 383 protein groups from control and treated samples. As many proteins were autoannotated as hypothetical proteins when identified against *Picocystis* database in Uniprot, the hypothetical proteins were subjected to NCBI protein BLAST. BLAST results having maximum coverage and lowest E-value for proteins identified were selected. Out of 243 identified hypothetical proteins, 197 proteins were annotated by NCBI BLAST algorithm. Detailed annotation information including peptide sequences, accession numbers, matching criteria, unused scores, *p*-value and sequence coverage of total identified and differential protein species is provided in Supplementary Table S1. Figure 1 depicts the overall changes in protein abundance in *P. salinarum* after 3 days of high DIC and salt treatment (MT). Of these total 383 proteins identified in the control and treatment, 257 proteins were common to both the conditions, and 119 were unique to the treated algal cells, while only 5 were unique to the control (Figure 1A; Supplementary Table S2). Of the 225 DAPs of the control and treated *P. salinarum*, a total of 150 DAPs were significantly altered in response to high DIC and salinity relative to control, with the threshold for upregulated expression, Log2 fold change >1 and downregulated expression Log2 fold change <−1 (*p* < 0.05). Seventy of these DAPs were upregulated and 64 were downregulated in the cells after 3 days salt treatment (Figure 1B; Supplementary Table S3). Details for each protein are also provided in Supplementary Table S1. Volcano plot shows the overall changes in protein abundance in treated compared to control cultures, with significant difference (*p* < 0.05) in the expression of a few proteins (Figure 1C). The log transformed abundance values were further Z scaled and used to plot Heatmap (Figure 1D; Supplementary Table S4). Supplementary Table S5 provides a combined tabulation of all results.

**Figure 2:**
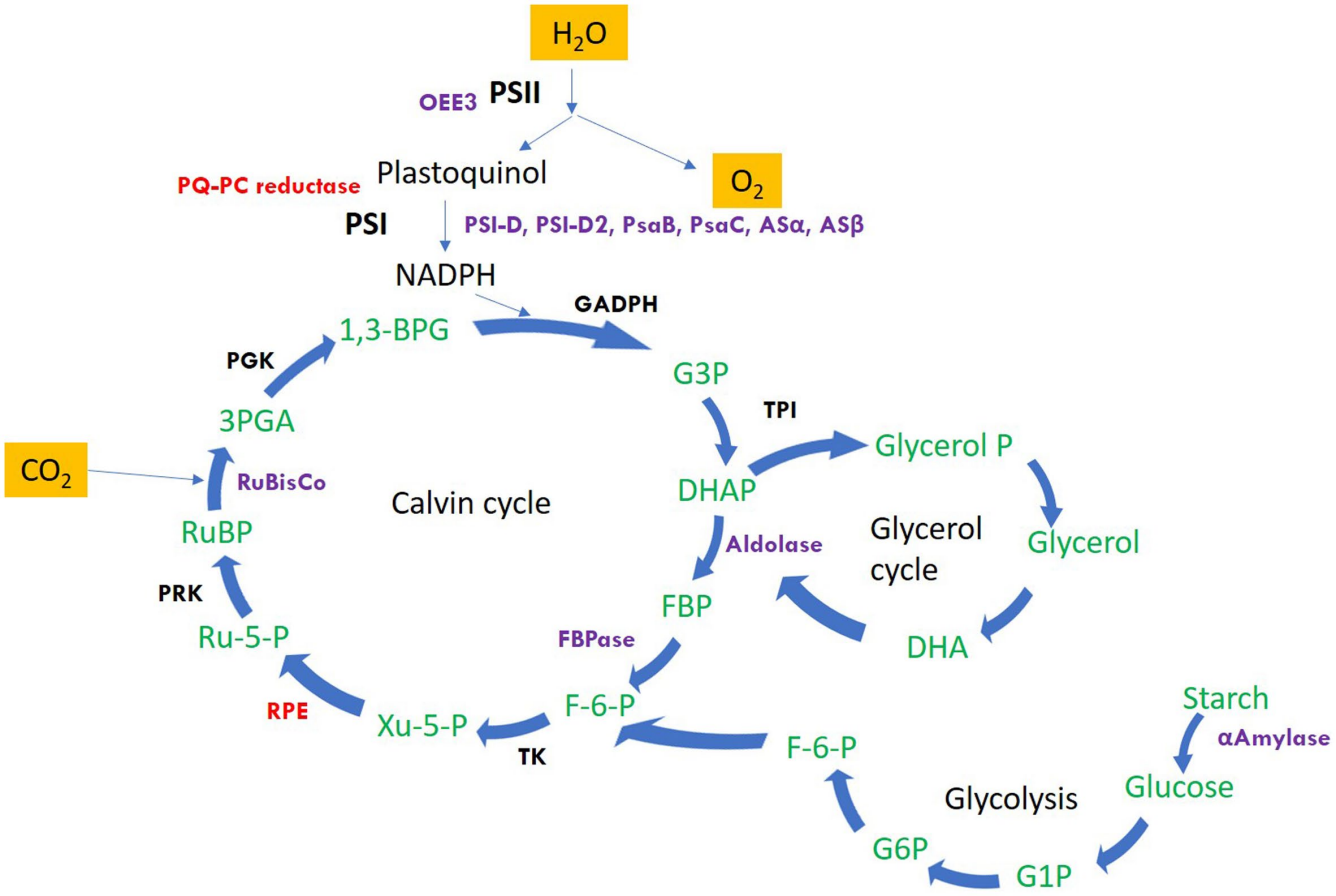
Schematic representation of the regulation of photosynthetic machinery and C assimilation in *P. salinarum* SLJS6 under highly saline alkaline conditions as per the results obtained in this study. Only the proteins that showed significant differential abundance are highlighted (Purple: significantly upregulated; Red: significantly down-regulated). Other proteins/enzymes of the depicted pathways were also differentially altered but were not significant (details of such proteins is given in Supplementary Tables). PSI-D, photosystem I reaction center subunit II; PSI-D2, photosystem I reaction center subunit II-2; PsaB, photosystem I P700 chlorophyll a apoprotein A1; PsaC, photosystem I iron-sulfur center; Asα, ATP synthase subunit alpha; Asβ, ATP synthase subunit beta; OEE3, oxygen-evolving enhancer protein 3; PQ-PC reductase, plastoquinone plastocyanin reductase; FBPase, fructose-1,6-bisphosphatase; FBA, fructose-bisphosphate aldolase; RuBisco, ribulose bisphosphate carboxylase; RPE, ribulosephosphate 3-epimerase; GAPDH, glyceraldehyde 3-phosphate dehydrogenase; G3P, glyceraldehyde 3-phosphate; TPI, triosephosphate isomerase; DHAP, dihydroxyacetone phosphate; FBP, fructose-1,6-bisphosphate; F-6-P, fructose-6-phosphate; Ru-5-P, ribulose 5-phosphate; PRK, phosphoribulokinase; RuBP, ribulose-1,5-bisphosphate; 3PGA, 3-phosphoglycerate; PGK, phosphoglycerate kinase; 1,3-BPG, 1,3-bisphosphoglycerate; TK, transketolase.

The significantly differentially altered proteins are involved in photosynthesis, calvin cycle, protein folding and refolding, glycolytic processes, gluconeogenesis, photosynthetic electron transport and photorespiration, carbohydrate metabolic process, protein catabolic process, proteolysis, cellular response to oxidative stress, and regulation of translation. The identified proteins along with the hypothetical proteins with their best blast match, categorized by their function and grouped as upregulated and downregulated are presented in Table 1. Figure 2 depicts the regulation of photosynthetic machinery and C assimilation in *P. salinarum* SLJS6 under highly saline alkaline conditions as per the results obtained in this study.

**Table 1:** Summary of protein changes in *P. salinarum* SLJS6 in response to high carbonates and NaCl concentrations (highly saline-alkaline environment).

Accession ID	Description	Fold change (FC)	log2_FC
**Upregulated**			
*Photosynthesis and C assimilation/metabolism*			
A0A088CIC9	Photosystem I P700 chlorophyll a apoprotein A1	4.93570353	2.30325574
A0A7S3XDW4	Photosystem I reaction center subunit II (PSI-D)	10.02954655	3.326184
A0A7S3UCD5	Photosystem I reaction center subunit II-2, (PSI-D2)	7.805584488	2.964507
A0A088CIC4	Photosystem I iron–sulfur center	4.60632224	2.20361534
A0A6U9QUQ2	Oxygen-evolving enhancer protein 3, (OEE3)	4.858613611	2.280545
A0A7S3UCI9	Ribulose bisphosphate carboxylase small subunit	2.13234392	1.09244014
A0A088CJ48	ATP synthase subunit b, chloroplastic	3.38476709	1.75905656
A0A6U9Q6Y4	Fructose-1,6-bisphosphatase class 2 (FBPase class 2)	32.52888111	5.023649
A0A7S3UEG8	Fructose-bisphosphate aldolase	14.01303947	3.808698
A0A7S3XC56	ATP synthase subunit beta	5.814289942	2.539603
A0A7S3XDI0	Acyl-CoA-binding protein (ACBP)	10.01742679	3.32444
A0A7S3U9Y0	Beta/alpha-amylase	4.742889258	2.245766
*Protein folding and integrity/chaperones*			
A0A7S3UAA2	Peptidylprolyl isomerase	14.33125	3.84109254
A0A7S3UDR2	Chaperonin CPN60-2, mitochondrial (HSP60-2)	3.94147883	1.978737
A0A7S3XCX1	Chaperonin CPN60-1, mitochondrial (HSP60-1)	3.753818827	1.908359
A0A7S3UFZ1	10 kDa chaperonin (chaperonin 10) (CPN10)	6.781891858	2.761688
A0A7S3XC49	GrpE protein homolog	2.60479811	1.38117156
*Energy*			
A0A7S3U9F1	ATP synthase subunit beta (fragment)	2.7153492	1.44113774
A0A7S3XC56	ATP synthase subunit beta	5.81428994	2.53960302
A0A7S3XCR9	ATP synthase subunit beta	5.81428994	2.53960302
A0A4D6C6K6	ATP synthase subunit alpha	4.14373407	2.05093142
*Translation*			
A0A7S3UH31	60S ribosomal protein L12-2	8.639568856	3.110959
A0A7S3UG40	40S ribosomal protein S26	7.75541268	2.95520355
A0A088CK85	50S ribosomal protein L14, chloroplastic	3.30604634	1.72510695
A0A088CJ84	50S ribosomal protein L5, chloroplastic	2.0786224	1.0556277
A0A7S3UFZ1	30S ribosomal protein S15	5.21668784	2.38313411
A0A7S3UFF6	Serine/arginine-rich splicing factor SR30	7.230565696	2.854109
*Amino-acid biosynthesis*			
A0A7S3UAB9	5-methyltetrahydropteroyltriglutamate--homocysteine S-methyltransferase	2.2874421	1.19373522
*Regulatory proteins and others*			
A0A7S3UCC8	GTP-binding nuclear protein	3.07584328	1.620982
A0A7S3U9A7	Plant UBX domain-containing protein 3 (PUX3)	5.090990276	2.347946
A0A7S3XCD1	Plant UBX domain-containing protein 4 (PUX4)	5.090990276	2.347946
A0A7S3UAA5	Protein DJ-1 homolog A (AtDJ1A)	4.247970072	2.086774
**Downregulated**			
*Translation*			
A0A088CJ89	30S ribosomal protein S9, chloroplastic	0.014067417	−6.151498764
A0A088CK88	50S ribosomal protein L32, chloroplastic	0.014158609	−6.14217661
A0A088CJ55	Translation initiation factor IF-1, chloroplastic	0.018108157	−5.787216448
*Carbohydrate metabolism*			
A0A7S3UAF0	Mannan endo-1,4-beta-mannosidase	0.145017641	−2.785699689
A0A7S3UAQ3	Alpha-1,4 glucan phosphorylase	0.187864537	−2.412235342
A0A7S3XDM6	Ribulose-phosphate 3-epimerase	0.397784619	−1.3299406
*Energy/phosphorylation*			
A0A6U9QFU4	Guanylate kinase	0.100303081	−3.317562176
*Photosynthesis*			
A0A7S3XBE2	Plastoquinol--plastocyanin reductase	0.203686059	−2.295580852
*Protein folding and integrity/chaperones*			
A0A7S3UAW4	Peptidylprolyl isomerase	0.216337686	−2.208643093

These differentially expressed proteins exhibited significant upregulation and downregulation (only selected representative DAPs are enlisted, for detailed information refer to Supplementary Tables.

## 4. Discussion

Sorokin et al., 2015 have described an emerging concept of ‘natronophily’ which states that the prokaryotic isolates from soda lakes can grow in almost pure sodium carbonate brines without needing high concentrations of chloride salts as required by haloalkaliphiles and better called as “natronophiles.” Life in soda brines is very different from life in NaCl brines due to amount the osmotic pressure put on living systems by concentrations of these two salts, former being a weak electrolyte than the later, therefore providing a strong energetic advantage over the life in salt brines, even with the extra energy demand for maintaining drastic pH gradient. *P. salinarum* SLJS6 was isolated from salt brine (NaCl 345 g/l, pH 10) of a hypersaline soda lake, Lake Sambhar in India. NaCl, Na_2_SO_4_, Na_2_CO_3_ and NaHCO_3_ make up to 98% constituents of the Sambhar lake brine (Sinha and Raymahashay, 2004). The chemical composition of lake Sambhar exhibiting low concentrations of divalent cations Ca^2+^ and Mg^2+^ is often compared and found similar to African soda lakes like lake Wadi Natrum and Lake Magadi (Upasani and Desai, 1990). Sambhar lake has been reported for halophilic archaea, bacteria and green algae exploring their biotechnological applications. Bacteria belonging to genus *Alteribacillus*, *Halobacillus*, *Halorubrum*, *Lentibacillus*, *Natronorubrum*, *Piscibacillus and Thalassobacillus* and archaea belonging to genus *Natronococcus, Natrialba*, *Halobiforma*, *Halostagnicola*, *Natronolimnobius*, *Natronobacterium*, *Haloterrigena*, *Halopiger*, *Halorubrum*, *Natrinema*, *Natronorubrum*, *Natronomonas*, *Haladaptatus* have been reported (Kajale et al., 2020). Most of the bacteria produce extremozymes (proteases, lipases, amylases, endoglucanase, endoxylanase etc.), bacteriocins, bioactive and antimicrobial compounds (Upasani and Desai, 1990; Sahay et al., 2012; Kajale et al., 2020). Among microalgae, cyanobacteria *Arthrospira* and green algae *Dunaliella* have received enormous scientific attention and have been widely characterized for various biotechnological applications (Phadwal and Singh, 2003; Kulshreshtha and Singh, 2013; Singh and Singhm, 2014; Singh et al., 2018; Vidyalaxmi et al., 2019; Ma et al., 2019; da Silva et al., 2021; Gonzalez and Laroche, 2021). Recent culture independent multi-omics study shed light on the microbiome evolution of lake Sambhar in comparison to other saline ecosystems, reported relative abundance of members of Archaea, Bacteria and Eukaryota domain, with *Dunaliella* well recognized under Chlorophyceae (Mehta et al., 2021), however, *P. salinarum* remains unreported. Next generation sequencing based culture independent studies have unveiled plethora of uncultured microorganisms, but culture-based studies are essential to discover new species, decipher physiological mechanisms and realize biotechnological potentials (Kajale et al., 2020). In light of this, an attempt was made to understand the molecular basis of the outstanding capabilities and physiology of the isolate SLJS6 growing under highly saline-alkaline conditions, through high throughput proteomics.

Of the 70 identified upregulated proteins, 46 proteins are hypothetical proteins, i.e., proteins that are predicted to be expressed from an open reading frame (ORF), but lack the experimental evidence of translation or the genome is just sequenced and full annotation has not been performed yet. *Picocystis* genomes have only been sequenced lately and only two genomes are available till date, whole genome shotgun sequence of *Picocystis* sp. ML (GenBank: QYZS01000302.1, release date: 16-Oct-2018) and *Picocystis* sp. L7 mitochondrion, complete sequence, whole genome shotgun sequence (GenBank: CM037205.1, release date: 24-Nov-2021). Both the strains were isolated from Mono Lake, California, United States. On account of scarce genome sequence availability and lack of annotation of proteins, most significantly expressed proteins came out to be hypothetical proteins. The hypothetical protein sequences were then analyzed using the protein BLAST by NCBI. BLAST results having maximum coverage and lowest E-value for proteins identified were selected. Out of 46 identified upregulated hypothetical proteins, 37 proteins were annotated by NCBI BLAST algorithm. In total, 61 individual DAPs with known function (present in several forms), significantly upregulated were identified. Similarly, out of the 64 significantly downregulated proteins, 42 could not be assigned a function, being hypothetical proteins. Out of 42 identified downregulated hypothetical proteins, 32 proteins were annotated by NCBI BLAST algorithm. A total of 54 individual DAPs with known function were significantly downregulated. The identified proteins along with the hypothetical proteins with their best blast match, categorized by their function and grouped as upregulated and downregulated are presented in Table 1. Based on functional annotation, the identified proteins can be majorly classified into 4 categories, i.e., (a) Photosynthesis, C assimilation and carbohydrate metabolism, (b) metabolic energy production, (c) protein folding and integrity/chaperones/stress response proteins and (d) translational machinery and other proteins (Table 1). Up-regulation of prime metabolic network in *P. salinarum* SLJS6 as revealed by the identified salinity-alkalinity induced proteins can explain the extraordinary ability of this alga to survive in highly saline-alkaline environments as soda lakes.

The first category comprises many forms of major components of the photosynthetic machinery and the Calvin cycle: As evident from the growth data from our previous study (Singh et al., 2022), the growth of *P. salinarum* SLJS6 under conditions of high alkalinity (high DIC) and salinity exhibited a sharp increase. Similarly, an overall surge in its cellular photosynthetic activity reflected by the highly upregulated photosynthetic proteins, could be due to the plentiful availability of inorganic C (in the form of HCO_3_^−^ and CO_3_^2−^) for carbon-fixation in M medium. Photosystem I reaction center subunit II (PSI-D), Photosystem I reaction center subunit II-2 (PSI-D2), Oxygen-evolving enhancer protein 3 (OEE3), Photosystem I P700 chlorophyll a apoprotein A1, Photosystem I iron–sulfur center, Ribulose bisphosphate carboxylase small subunit, ATP synthase subunit b, ATP synthase subunit beta, ATP synthase subunit alpha, Fructose-1,6-bisphosphatase, Fructose-bisphosphate aldolase, all these proteins were highly upregulated, with some forms showing abundance more than 10 folds in the M medium grown cells. Figure 2 depicts the regulation of photosynthetic machinery and C assimilation in *P. salinarum* SLJS6 under highly saline alkaline conditions as per the results obtained in this study. Photosynthesis comprises a series of electron transport reactions in the thylakoid membrane and carbon fixation reactions in the stroma. In the thylakoid reactions, water is split and electrons excised in PSII are transferred to NADP^+^ through the cytochrome *b*_6_*f* complex and PSI, thereby producing NADPH. This linear transport of electron is coupled with the translocation of protons across the thylakoid membrane from the stroma to the lumen. ATP is then synthesized utilizing the generated proton motive force. NADPH and ATP in turn are used to fix inorganic carbon in the Calvin-Benson cycle. In addition to this linear electron transport from water to NADP^+^, a cyclic electron transport operates at PSI that contributes to the supply of ATP for carbon fixation (Okegawa et al., 2020). PSI cyclic electron transport is essential for photosynthesis as well as photoprotection (Munekage et al., 2004). The significantly increased abundances of PSI proteins in our treated samples suggest that the cells must have induced the cyclic electron transport chain to produce more ATP that could be used to fix more of inorganic carbon, corroborated by in tandem increase in ATP synthase subunits beta and alpha. Another very crucial component of photosynthetic machinery that was highly abundant is oxygen-evolving enhancer protein 3 (OEE 3). The PSII oxygen-evolving complex (OEC) oxidizes water to provide protons for use by PSI, and consists of OEE1 (PsbO), OEE2 (PsbP) and OEE3 (PsbQ)^2^. The overexpression of OEE3 also points towards increased photosynthesis. It is implied that the photosynthetic Carbon assimilation was increased in *P. salinarum* grown in M medium under high DIC and salinity conditions. In previous experiments conducted for assessing the prospects of isolate SLJS6 as promising candidate for bicarbonate-based biomass production and carbon capture, we found that the growth rate (0.2282 d^−1^) and biomass productivity (0.498 g/l/d) of *P. salinarum* was increased many folds (>35) in M medium compared to BG11 medium (Singh et al., 2022). The identified induced proteins in *P. salinarum* in response to high DIC (bicarbonate/carbonate alkalinity) and salinity (NaCl) suggest enhanced photosynthesis and remarkable osmoadaptation at expense of energy and provide insights into the extraordinary ability of this haloalkaliphilic picoalga to flourish in extreme conditions, corroborating to its occurrence in extremely saline-alkaline environments around the world, outcompeting the major primary producers at highest salinities (Krienitz and Kotut, 2010; Krienitz, 2018; Pálmai et al., 2020). Studies have reported similar outcomes for *Dunaliella* which is extensively studied and appreciated for its halotolerance. Most freshwater and marine microalgae as well as plants show decreased photosynthetic activity in response to salt stress (Wingler et al., 2000; Neelam and Subramanyam, 2013). In that case this innate ability of *P. salinarum* to enhance photosynthesis is remarkable and must be subjected to intensive exploration. Two of the most overexpressed and very important proteins in this category are enzymes Fructose-1,6-bisphosphate aldolase (FBA) (fold change >32) and fructose-1,6-bisphosphatase class 2 (FBPase), fold change >12. Fructose-1,6-bisphosphatase class 2/sedoheputulose-1,7-bisphosphatase (FBPase class-2/SBPase) specifically those from algae are bi functional and can catalyzes the hydrolysis of fructose 1,6-bisphosphate and sedoheptulose 1,7-bisphosphate to fructose 6-phosphate and sedoheptulose 7-phosphate, respectively and are key regulator in Gluconeogenesis. They exist at a point in the Calvin cycle where the assimilated C could either be directed towards regeneration of CO_2_ acceptor molecule ribulose-1,5-bisphosphate (RuBP) to continue the cycle or to go into starch production (Miyagawa et al., 2001). Overexpression of cyanobacterial FBPase class-2/SBPase in tobacco plants, *Arabidopsis*, soybeans, and the oleaginous microalgae *Eugrena gracilis*, led to enhanced photosynthesis and biomass production (Miyagawa et al., 2001; Rosenthal et al., 2011; Ogawa et al., 2015; Köhler et al., 2016; Otori et al., 2017; De Porcellinis et al., 2018). Likewise, augmented levels of FBPase class-2/SBPase in our study supports the increased biomass production by *P. salinarum* SLJS6 in M medium. The other enzyme FBA catalyzes a reversible conversion of fructose-1,6-bisphosphate to glyceraldehyde-3-phosphate (G3P) and dihydroxyacetone phosphate (DHAP) in glycolysis, gluconeogenesis, and the Calvin-Benson cycle (Lee et al., 2020). In the proteome of *P. salinarum* SLJS6 grown in M medium, FBPase/SBPase and FBA were found to be predominantly overexpressed. Akin to increased abundance of FBPase/SBPase, high abundance of FBA also implies augmented C fixation in treated cells. High growth rate and biomass productivity of SLJS6 in M medium could be thus attributed to abundance of these two proteins along with upregulated components photosynthetic machinery. Moreover, FBA also has a significant role in the glycerol cycle, DHAP produced by the action of FBA could be converted to Glycerol (Liska et al., 2004). Liska et al. (2004) hypothesized that photosynthesis and energy utilization is enhanced in *Dunaliella* in highly saline conditions in order to enable massive production of glycerol. *Dunaliella* is known to prefer glycerol as an osmolyte (Kirst, 1989; Gunde-Cimerman et al., 2018). However, there are no reports on glycerol production in *Picocystis* so far. Nonetheless, *Picocystis* strain ML from Mono Lake was observed to grow over salinity range of 0–260 ‰, and was reported to contain dimethylsulfoniopropionate (DMSP) and Glycine Betaine (GB) as osmolytes (Roesler et al., 2002). The preference for high ionic strength is possibly backed up by some specialized metabolic regulations of cellular osmolarity. Some prokaryotic as well as eukaryotic microorganisms use a “low-salt in” strategy, wherein they accumulate organic osmotic, “compatible” solutes. Such microorganisms are very versatile in terms of salinity tolerance, and they do so at an expense of energy (Kirst, 1989). In this study, overexpression of FBA in treated samples could be assumed to be associated with glycerol production and accumulation as an osmolyte, but this must be ascertained by further experimental evidence.

Enzymes involved in the generation of metabolic energy (ATP and redox equivalents) were highly upregulated. Alpha and beta subunits of ATP synthase (both chloroplastic and mitochondrial) were expressed by more than 4 folds in the M medium grown algal cells, suggesting that extremophile *P. salinarum* cells produced extra metabolic energy to maintain normal biological functions. The metabolic energy for enhanced Carbon assimilation, glycerol production and ion transport is supplied by increased ATP and redox equivalent production (Katz and Pick, 2001). As discussed earlier increased NADPH and ATP produced as a result of enhanced photosynthetic light reaction are used to fix inorganic carbon in the Calvin-Benson cycle (Munekage et al., 2004; Okegawa et al., 2020). Moreover, many ATP dependent chaperon proteins function under high saline-alkaline conditions to maintain the structural integrity of proteins enabling their proper biological functioning, which is discussed in detail next.

Osmotic stress can disrupt cellular protein integrity and homeostasis by increasing the rate of protein unfolding, misfolding. The third major category of upregulated proteins was of Chaperone proteins/heat shock proteins that have an important role in maintaining the protein structural integrity of other proteins by folding and repairing misfolding (Zhang et al., 2016; Rutgers et al., 2017). In the case of *P. salinarum*, enzyme Peptidylprolyl isomerase, also known as cyclophilin A was highly overexpressed (>12 folds). Peptidyl-prolyl cis–trans isomerases (PPIases) are the only class of enzymes capable of cis–trans isomerization of the prolyl peptide bond, a rate limiting step in protein folding (Fischer and Bang, 1985; Singh et al., 2021). Along with PPIases, GrpE proteins were also found to be significantly upregulated. GrpE proteins actively contribute to the response to hyperosmotic and heat shock by preventing the aggregation of stress-denatured proteins. It is the nucleotide exchange factor for DnaK and may function as a thermosensor. Unfolded proteins bind initially to DnaJ; upon interaction with the DnaJ-bound protein, DnaK hydrolyzes its bound ATP, resulting in the formation of a stable complex. GrpE releases ADP from DnaK; ATP binding to DnaK triggers the release of the substrate protein, thus completing the reaction cycle. Several rounds of ATP-dependent interactions between DnaJ, DnaK and GrpE are required for fully efficient folding (Wu et al., 1996; Brehmer et al., 2004). Thus, it is ascertained that *P. salinarum* maintains its cellular protein structural integrity even in high osmotic stress by highly overexpressing such chaperone proteins. Mitochondrial Chaperonin CPN60-2, CPN60-1 and 10 kDa chaperonin (CPN10) were also significantly upregulated. Similarly, *D. salina* has been reported to regulate expression of heat shock proteins like HSP70B and HSP90A to alleviate salinity stress (Katz et al., 2007; Wang et al., 2019). Also, it corroborates with the increased production of energy currencies of the cell which are required to carry out such cascades of reactions important in maintaining the protein functional structure. Protein disulfide isomerase (PDI, EC 5.3.4.1) is another abundant upregulated protein that catalyzes disulfide bond formation and rearrangement in newly synthesized polypeptides and also acts as a molecular chaperone (Freedman and Klappa, 1999). Other upregulated enzymes identified are 5-methyltetrahydropteroyltriglutamate--homocysteine S-methyltransferase and GTP-binding nuclear protein. 5 methyltetrahydropteroyltriglutamate-homocysteine S-methyltransferase is involved in Amino acid biosynthesis (methionine biosynthesis) and has been reported to be overexpressed in plants in response to salt stress (Guo et al., 2019).

On the other hand, the majorly downregulated proteins were proteins belonging to the Translational machinery of the cell. Several forms of 30S, 50S, 40S and 60S ribosomal protein were significantly downregulated. Nevertheless, many were significantly upregulated too, but the downregulation was more pronounced. Translational machinery acquires new components depending on the environmental perturbations to which organisms are exposed, and the regulation of translation might be tailored in accordance with the specific requirements of the cells (Tiller and Bock, 2014). An interesting finding is that significant group of proteins, i.e., plant ubiquitin regulatory X domain-containing proteins: Plant UBX domain-containing protein 3 (PUX3) and plant UBX domain-containing protein 4 (PUX4) were also found to be highly upregulated in M medium grown cells. These PUXs are involved in regulation of the cell division control protein 48 (CDC48) that forms the core of a multifunctional multiprotein complex that can control protein/RNA expression and extract proteins from their environment for reuse or degradation (Zhang et al., 2021). Also, another regulatory protein AtDJ1C, belonging to the DJ-1 superfamily has been found to have essential role in chloroplast development and maturation in *Arabidopsis thaliana* (Lin et al., 2011). This protein also showed a marked up-regulation in samples grown in M medium. Thus, translational regulation in *P. salinarum* seems rather complex and would need further research and analyses. The other significantly downregulated proteins identified were related to carbohydrate metabolism including Mannan endo-1,4-beta-mannosidase, alpha-1,4 glucan phosphorylase and ribulose-phosphate 3-epimerase. Mannan endo-1,4-beta-mannosidase, commonly known β-mannanases is one of the main hydrolytic enzymes hydrolysing the mannan polymer thereby loosening and remodelling tough cell walls. Mannan rich cell walls are more flexible and extensible. Mannan also serves as storage polysaccharide (Rodríguez-Gacio et al., 2012). The downregulation of mannan endo-1,4-beta-mannosidase may be linked to maintaining cell wall flexibility during osmotic response. Alpha-1,4 glucan phosphorylase is mainly involved in starch degradation. *Chlamydomonas reinhardtii* has been previously reported to accumulate carbohydrate as starch in response to physiological stress (Longworth et al., 2012) resulting in storage of assimilated carbon that could be later used in biosynthesis of Triglycerides (Pick and Avidan, 2017).

By and large, our findings suggest that the haloalkaliphilic picoalga *P. salinarum* SLJS6 is endowed with innate complex mechanisms to adapt to highly saline-alkaline conditions and based on the knowledge acquired heretofore, it involves up-regulation of proteins and enzymes mainly involved in photosynthesis and carbon assimilation, ATP generation and protein folding-refolding. This study provided the first insights into the proteome of extremophilic alga *P. salinarum* exhibiting tailored regulatory mechanism of osmotic adaptation and proliferation in polyextreme conditions prevailing in saline sodic ecosystems, potentially unraveling the basis of resilience in this not so known organism and paves the way for a promising future candidate for biotechnological applications and model organism for deciphering the molecular mechanisms of osmotic adaptation. Further research on this unique photosynthetic organism is vital.

## Data availability statement

The datasets presented in this study can be found in online repositories. The names of the repository/repositories and accession number(s) can be found below: ProteomeXchange Consortium via the PRIDE - PXD037170.

## Author contributions

JS conceived, designed and performed the experiments and drafted and revised the manuscript. CM helped in sampling and performed chemical analysis related to this work. SK analyzed the proteome data. GJ and DD provided the infrastructure required for the work. All authors contributed to the article and approved the submitted version.

## Funding

This work was supported by grants from Department of Science and Technology SERB-NPDF (PDF/2015/001067) and INSPIRE faculty Fellowship (DST/INSPIRE/04/2018/002952), Government of India (GoI), New Delhi, India.

## Conflict of interest

SK and GJ were employed by Vproteomics, Valerian Chem Private Limited, New Delhi, India.

The remaining authors declare that the research was conducted in the absence of any commercial or financial relationships that could be construed as a potential conflict of interest.

## Publisher’s note

All claims expressed in this article are solely those of the authors and do not necessarily represent those of their affiliated organizations, or those of the publisher, the editors and the reviewers. Any product that may be evaluated in this article, or claim that may be made by its manufacturer, is not guaranteed or endorsed by the publisher.

## Footnotes

^1^https://www.uniprot.org/uniprotkb?query=Picocystis%20salinarum

^2^https://www.ebi.ac.uk/interpro/entry/InterPro/IPR008797/
